# 2-Fluoro-6-[(*E*)-(pyridin-2-yl)imino­meth­yl]phenol

**DOI:** 10.1107/S1600536811048963

**Published:** 2011-11-23

**Authors:** Rui-Qin Fang, Tao Song, Min-Min Shi

**Affiliations:** a School of Life Science and Technology, University of Electronic Science and Technology of China, Chengdu 610054, People’s Republic of China; b State Key Laboratory of Pharmaceutical Biotechnology, Nanjing University, Nanjing 210093, People’s Republic of China

## Abstract

The title compound, C_12_H_9_FN_2_O, is almost planar (r.m.s. deviation for the 16 non-H atoms = 0.019 Å), a conformation stabilized by an intra­molecular O—H⋯N hydrogen bond, which generates an *S*(6) ring. In the crystal, inversion dimers linked by pairs of C—H⋯O hydrogen bonds generate *R*_2_^2^(16) loops.

## Related literature

For a related structure, see: Cui & Shi (2009 ▶).
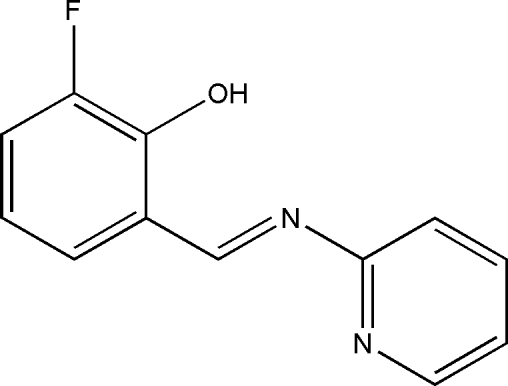


## Experimental

### 

#### Crystal data

C_12_H_9_FN_2_O
*M* *_r_* = 216.21
Monoclinic, 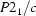

*a* = 5.012 (1) Å
*b* = 19.764 (4) Å
*c* = 10.802 (2) Å
β = 101.42 (3)°
*V* = 1048.8 (4) Å^3^
*Z* = 4
Mo *K*α radiation
μ = 0.10 mm^−1^
*T* = 293 K
0.29 × 0.22 × 0.18 mm

#### Data collection

Enraf–Nonius CAD-4 diffractometer
Absorption correction: ψ scan (North *et al.*, 1968) ▶ *T*_min_ = 0.971, *T*_max_ = 0.982
2284 measured reflections
2047 independent reflections
884 reflections with *I* > 2σ(*I*)
*R*_int_ = 0.027

#### Refinement

*R*[*F* ^2^ > 2σ(*F* ^2^)] = 0.069
*wR*(*F* ^2^) = 0.177
*S* = 1.03
2047 reflections
145 parameters
H-atom parameters constrained
Δρ_max_ = 0.15 e Å^−3^
Δρ_min_ = −0.18 e Å^−3^

### 

Data collection: *CAD-4 Software* (Enraf–Nonius, 1989 ▶); cell refinement: *CAD-4 Software*; data reduction: *XCAD4* (Harms & Wocadlo, 1995 ▶); program(s) used to solve structure: *SHELXS97* (Sheldrick, 2008 ▶); program(s) used to refine structure: *SHELXL97* (Sheldrick, 2008 ▶); molecular graphics: *SHELXTL* (Sheldrick, 2008 ▶); software used to prepare material for publication: *SHELXTL*.

## Supplementary Material

Crystal structure: contains datablock(s) global, I. DOI: 10.1107/S1600536811048963/hb6518sup1.cif

Supplementary material file. DOI: 10.1107/S1600536811048963/hb6518Isup2.cdx

Structure factors: contains datablock(s) I. DOI: 10.1107/S1600536811048963/hb6518Isup3.hkl

Supplementary material file. DOI: 10.1107/S1600536811048963/hb6518Isup4.cml

Additional supplementary materials:  crystallographic information; 3D view; checkCIF report

## Figures and Tables

**Table 1:** Hydrogen-bond geometry (Å, °)

*D*—H⋯*A*	*D*—H	H⋯*A*	*D*⋯*A*	*D*—H⋯*A*
O1—H1⋯N1	0.82	1.88	2.586 (4)	144
C9—H9⋯O1^i^	0.93	2.60	3.390 (5)	143

Symmetry code: (i) 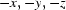.

**Fig. 1.**
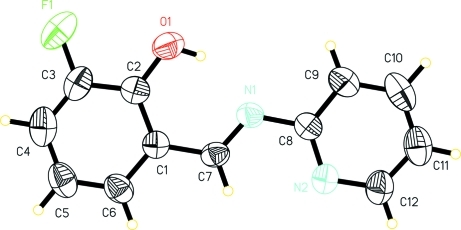
The structure of the title compound (I) showing 35% probability displacement ellipsoids.

**Fig. 2.**
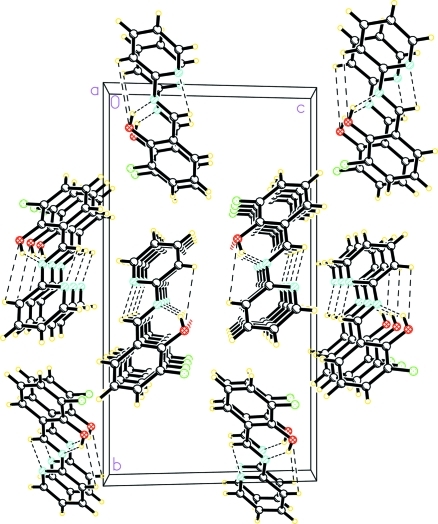
The crystal packing of (I), viewed along the a axis. Hydrogen bonds are shown as dashed lines.

## supplementary crystallographic information

### Comment

The crystal structrue of 4-fluoro-2-[(*E*)-2-pyridyliminomethyl]phenol has been reported before, which is synthesized by 5-fluoro-salicyladehyde and pyridin-2-amine, and the distinction between title compound (I) and 4-fluoro-2-[(E)-2-pyridyliminomethyl]phenol are the different substituent position of the fluoro substituent (Cui *et al.*, 2009).

The molecular structure of title compound is shown in Fig. 1, and selected geometric parameters are listed in Table 1. The C—F single bond length is 1.366 (4) Å, the C—O single bond length is 1.330 (4) Å, and the C—N double bond length is 1.273 (4) Å, which is similar with the bond lengths of reference compound (Cui *et al.*, 2009). The benzene and pyridine, of course, planar with the Rms deviation of 0.0082 and 0.0015 Å, respectively. Atom F1 and O1 are almost on the benzene plane displaced by -0.0164 (49) and 0.0220 (45) Å, and N1 attached to prydine plane deviated slightly with the distance of 0.0115 (47) Å. The dihedral angle between benzene and pyridine in title compound is 1.14 (23) °, which is smaller than that in 4-Fluoro-2-[(*E*)-2-pyridyliminomethyl]phenol. Intramolecular O—H···N hydrogen bonds and intermolecular C—H···O links occur (Table 2), and these lead to packing network of the molecules (Fig. 2).

### Experimental

The title compound was prepared by stirring a mixture of 3-fluoro-salicylaldehyde (140 mg, 1 mmol) and pyridin-2-amine (94 mg, 1 mmol) in methanol (15 ml) for 3 h at room temperature. After keeping the solution in air for 5 d, yellow block-shaped crystals of (I) were formed. The crystals were isolated, washed three times with methanol and dried in a vacuum desiccator containing anhydrous CaCl_2_.

### Refinement

All the H atoms, were placed in idealized positions (C—H = 0.93- 0.96 Å, O—H = 0.82 Å) and refined as riding with *U*_iso_(H) = 1.2*U*_eq_(C) and *U*_iso_(H) = 1.5*U*_eq_(O).

### Crystal data

**Table:**

C_12_H_9_FN_2_O	*F*(000) = 448
*M**_r_* = 216.21	*D*_x_ = 1.369 Mg m^−^^3^
Monoclinic, *P*2_1_/*c*	Mo *K*α radiation, λ = 0.71073 Å
Hall symbol: -P 2ybc	Cell parameters from 763 reflections
*a* = 5.012 (1) Å	θ = 3.2–24.2°
*b* = 19.764 (4) Å	µ = 0.10 mm^−^^1^
*c* = 10.802 (2) Å	*T* = 293 K
β = 101.42 (3)°	Block, yellow
*V* = 1048.8 (4) Å^3^	0.29 × 0.22 × 0.18 mm
*Z* = 4	

### Data collection

**Table:**

Enraf–Nonius CAD-4 diffractometer	2047 independent reflections
Radiation source: fine-focus sealed tube	884 reflections with *I* > 2σ(*I*)
graphite	*R*_int_ = 0.027
ω/2θ scan	θ_max_ = 26.0°, θ_min_ = 2.1°
Absorption correction: ψ scan (North *et al.*, 1968)	*h* = 0→6
*T*_min_ = 0.971, *T*_max_ = 0.982	*k* = 0→24
2284 measured reflections	*l* = −13→13

### Refinement

**Table:**

Refinement on *F*^2^	Primary atom site location: structure-invariant direct methods
Least-squares matrix: full	Secondary atom site location: difference Fourier map
*R*[*F*^2^ > 2σ(*F*^2^)] = 0.069	Hydrogen site location: inferred from neighbouring sites
*wR*(*F*^2^) = 0.177	H-atom parameters constrained
*S* = 1.03	*w* = 1/[σ^2^(*F*_o_^2^) + (0.0605*P*)^2^] where *P* = (*F*_o_^2^ + 2*F*_c_^2^)/3
2047 reflections	(Δ/σ)_max_ < 0.001
145 parameters	Δρ_max_ = 0.15 e Å^−^^3^
0 restraints	Δρ_min_ = −0.18 e Å^−^^3^

### Special details

**Table:**

Geometry. All e.s.d.'s (except the e.s.d. in the dihedral angle between two l.s. planes) are estimated using the full covariance matrix. The cell e.s.d.'s are taken into account individually in the estimation of e.s.d.'s in distances, angles and torsion angles; correlations between e.s.d.'s in cell parameters are only used when they are defined by crystal symmetry. An approximate (isotropic) treatment of cell e.s.d.'s is used for estimating e.s.d.'s involving l.s. planes.
Refinement. Refinement of *F*^2^ against ALL reflections. The weighted *R*-factor *wR* and goodness of fit *S* are based on *F*^2^, conventional *R*-factors *R* are based on *F*, with *F* set to zero for negative *F*^2^. The threshold expression of *F*^2^ > σ(*F*^2^) is used only for calculating *R*-factors(gt) *etc*. and is not relevant to the choice of reflections for refinement. *R*-factors based on *F*^2^ are statistically about twice as large as those based on *F*, and *R*- factors based on ALL data will be even larger.

### Fractional atomic coordinates and isotropic or equivalent isotropic displacement parameters (Å^2^)

**Table:**

	*x*	*y*	*z*	*U*_iso_*/*U*_eq_	
C1	0.1221 (7)	0.14229 (17)	0.3242 (3)	0.0587 (9)	
C2	−0.0882 (7)	0.14860 (18)	0.2172 (3)	0.0646 (10)	
C3	−0.2793 (8)	0.1990 (2)	0.2189 (4)	0.0782 (11)	
C4	−0.2688 (9)	0.2415 (2)	0.3172 (5)	0.0885 (13)	
H4	−0.4033	0.2741	0.3153	0.106*	
C5	−0.0592 (9)	0.2366 (2)	0.4204 (5)	0.0871 (13)	
H5	−0.0473	0.2670	0.4871	0.104*	
C6	0.1307 (8)	0.18699 (18)	0.4240 (4)	0.0744 (11)	
H6	0.2692	0.1829	0.4950	0.089*	
C7	0.3252 (7)	0.08971 (17)	0.3301 (3)	0.0623 (10)	
H7	0.4609	0.0860	0.4022	0.075*	
C8	0.5244 (8)	−0.00312 (17)	0.2485 (3)	0.0604 (9)	
C9	0.5107 (9)	−0.0465 (2)	0.1482 (4)	0.0800 (11)	
H9	0.3724	−0.0424	0.0772	0.096*	
C10	0.7053 (11)	−0.0961 (2)	0.1548 (5)	0.0976 (15)	
H10	0.7021	−0.1261	0.0880	0.117*	
C11	0.9045 (10)	−0.1006 (2)	0.2620 (5)	0.0955 (14)	
H11	1.0381	−0.1338	0.2696	0.115*	
C12	0.9025 (8)	−0.0553 (2)	0.3571 (4)	0.0849 (12)	
H12	1.0389	−0.0586	0.4290	0.102*	
F1	−0.4856 (5)	0.20355 (13)	0.1156 (2)	0.1162 (10)	
N1	0.3236 (5)	0.04836 (14)	0.2396 (2)	0.0614 (8)	
N2	0.7175 (7)	−0.00676 (15)	0.3531 (3)	0.0711 (9)	
O1	−0.1081 (5)	0.10814 (13)	0.1174 (2)	0.0838 (8)	
H1	−0.0026	0.0763	0.1347	0.126*	

### Atomic displacement parameters (Å^2^)

**Table:**

	*U*^11^	*U*^22^	*U*^33^	*U*^12^	*U*^13^	*U*^23^
C1	0.053 (2)	0.059 (2)	0.067 (2)	−0.0073 (19)	0.0176 (19)	0.0026 (18)
C2	0.064 (3)	0.063 (2)	0.068 (2)	−0.013 (2)	0.016 (2)	0.001 (2)
C3	0.062 (3)	0.072 (3)	0.101 (3)	0.002 (2)	0.019 (2)	0.023 (3)
C4	0.071 (3)	0.068 (3)	0.136 (4)	0.002 (2)	0.046 (3)	0.011 (3)
C5	0.081 (3)	0.075 (3)	0.115 (4)	−0.008 (3)	0.041 (3)	−0.021 (3)
C6	0.071 (3)	0.071 (3)	0.084 (3)	−0.013 (2)	0.021 (2)	−0.016 (2)
C7	0.058 (2)	0.062 (2)	0.063 (2)	−0.0091 (19)	0.0022 (18)	0.0014 (19)
C8	0.067 (2)	0.053 (2)	0.063 (2)	−0.0106 (19)	0.018 (2)	−0.0006 (19)
C9	0.089 (3)	0.077 (3)	0.077 (3)	−0.011 (3)	0.023 (2)	−0.014 (2)
C10	0.132 (4)	0.068 (3)	0.109 (4)	−0.008 (3)	0.062 (4)	−0.018 (3)
C11	0.101 (4)	0.067 (3)	0.129 (4)	0.005 (3)	0.049 (3)	−0.001 (3)
C12	0.079 (3)	0.070 (3)	0.108 (3)	0.007 (2)	0.023 (3)	0.002 (3)
F1	0.0764 (16)	0.136 (2)	0.129 (2)	0.0213 (15)	0.0034 (15)	0.0401 (17)
N1	0.0580 (18)	0.0615 (18)	0.0625 (18)	−0.0073 (16)	0.0064 (15)	−0.0075 (16)
N2	0.068 (2)	0.067 (2)	0.079 (2)	0.0056 (17)	0.0168 (18)	0.0019 (17)
O1	0.0758 (18)	0.0932 (19)	0.0748 (17)	0.0018 (15)	−0.0033 (14)	0.0013 (16)

### Geometric parameters (Å, °)

**Table:**

C1—C6	1.388 (4)	C7—H7	0.9300
C1—C2	1.407 (5)	C8—N2	1.336 (4)
C1—C7	1.447 (4)	C8—C9	1.372 (5)
C2—O1	1.330 (4)	C8—N1	1.421 (4)
C2—C3	1.385 (5)	C9—C10	1.375 (5)
C3—C4	1.346 (5)	C9—H9	0.9300
C3—F1	1.366 (4)	C10—C11	1.374 (6)
C4—C5	1.375 (5)	C10—H10	0.9300
C4—H4	0.9300	C11—C12	1.364 (5)
C5—C6	1.362 (5)	C11—H11	0.9300
C5—H5	0.9300	C12—N2	1.329 (4)
C6—H6	0.9300	C12—H12	0.9300
C7—N1	1.273 (4)	O1—H1	0.8186
			
C6—C1—C2	119.0 (3)	C1—C7—H7	119.0
C6—C1—C7	120.6 (4)	N2—C8—C9	123.3 (4)
C2—C1—C7	120.4 (3)	N2—C8—N1	118.7 (3)
O1—C2—C3	120.2 (4)	C9—C8—N1	118.0 (4)
O1—C2—C1	122.4 (3)	C8—C9—C10	118.8 (4)
C3—C2—C1	117.4 (4)	C8—C9—H9	120.6
C4—C3—F1	120.6 (4)	C10—C9—H9	120.6
C4—C3—C2	122.7 (4)	C11—C10—C9	118.6 (4)
F1—C3—C2	116.7 (4)	C11—C10—H10	120.7
C3—C4—C5	120.0 (4)	C9—C10—H10	120.7
C3—C4—H4	120.0	C12—C11—C10	118.7 (4)
C5—C4—H4	120.0	C12—C11—H11	120.6
C6—C5—C4	119.4 (4)	C10—C11—H11	120.6
C6—C5—H5	120.3	N2—C12—C11	124.0 (4)
C4—C5—H5	120.3	N2—C12—H12	118.0
C5—C6—C1	121.5 (4)	C11—C12—H12	118.0
C5—C6—H6	119.3	C7—N1—C8	120.7 (3)
C1—C6—H6	119.3	C12—N2—C8	116.7 (3)
N1—C7—C1	122.0 (3)	C2—O1—H1	109.5
N1—C7—H7	119.0		
			
C6—C1—C2—O1	179.3 (3)	C6—C1—C7—N1	−179.5 (3)
C7—C1—C2—O1	−1.1 (5)	C2—C1—C7—N1	0.9 (5)
C6—C1—C2—C3	−1.0 (5)	N2—C8—C9—C10	−0.3 (5)
C7—C1—C2—C3	178.6 (3)	N1—C8—C9—C10	179.3 (3)
O1—C2—C3—C4	−179.8 (3)	C8—C9—C10—C11	0.5 (6)
C1—C2—C3—C4	0.4 (5)	C9—C10—C11—C12	−0.5 (6)
O1—C2—C3—F1	1.1 (5)	C10—C11—C12—N2	0.4 (6)
C1—C2—C3—F1	−178.6 (3)	C1—C7—N1—C8	−179.5 (3)
F1—C3—C4—C5	−179.7 (3)	N2—C8—N1—C7	−0.9 (5)
C2—C3—C4—C5	1.3 (6)	C9—C8—N1—C7	179.5 (3)
C3—C4—C5—C6	−2.5 (6)	C11—C12—N2—C8	−0.2 (6)
C4—C5—C6—C1	1.9 (6)	C9—C8—N2—C12	0.1 (5)
C2—C1—C6—C5	−0.1 (5)	N1—C8—N2—C12	−179.5 (3)
C7—C1—C6—C5	−179.7 (3)		

### Hydrogen-bond geometry (Å, °)

**Table:**

*D*—H···*A*	*D*—H	H···*A*	*D*···*A*	*D*—H···*A*
O1—H1···N1	0.82	1.88	2.586 (4)	144
C9—H9···O1^i^	0.93	2.60	3.390 (5)	143

Symmetry codes: (i) −*x*, −*y*, −*z*.
